# Striving to Avoid Inferiority and Procrastination among University Students: The Mediating Roles of Stress and Self-Control

**DOI:** 10.3390/ijerph18115570

**Published:** 2021-05-23

**Authors:** Peter-Yee-Lap To, Barbara-Chuen-Yee Lo, Ting-Kin Ng, Bernard-Pak-Ho Wong, Anna-Wai-Man Choi

**Affiliations:** 1 Department of Applied Psychology, Lingnan University, Hong Kong, China; yeelapto@ln.hk; 2 Wofoo Joseph Lee Consulting and Counselling Psychology Research Centre, Lingnan University, Hong Kong, China; ngtingkin@gmail.com; 3 Department of Educational Psychology, The Chinese University of Hong Kong, Hong Kong, China; bphwong@cuhk.edu.hk; 4 Department of Social and Behavioural Sciences, City University of Hong Kong, Hong Kong, China; anna.choi@cityu.edu.hk

**Keywords:** procrastination, striving to avoid inferiority, performance-avoidance goal orientation, stress, self-control

## Abstract

The current study intended to examine whether the relationship between university students’ striving to avoid inferiority (SAI) and procrastination was serially mediated by stress and self-control. The sample consisted of 154 Hong Kong university students. Their levels of striving to avoid inferiority, stress, self-control, and procrastination were measured by the *Striving to Avoid Inferiority Scale (SAIS)*, the stress subscale of the Depression Anxiety Stress Scales (DASS-21), the Short Self-Regulation Questionnaire (SSRQ), and the General Procrastination Scale (GPS), respectively. The results of structural equation modeling revealed that SAI positively predicted stress, stress negatively predicted self-control, and self-control negatively predicted procrastination. SAI did not directly predict procrastination. The results of bootstrapping analyses supported the hypotheses that the effect of stress on procrastination was mediated by self-control, the effect of SAI on self-control was mediated by stress, and more importantly, the effect of SAI on procrastination was serially mediated by stress and self-control. Further research is suggested to investigate the thoughts and feelings pertinent to procrastination and the actual duration of procrastination among university students.

## 1. Introduction

Defined as the tendency to delay the task initiation or completion despite expecting to be worse off for doing so, procrastination has received increasing research attention in recent decades [1]. The struggle to prevent procrastinating behaviors has been one of the major difficulties experienced by nearly all students. The rise in research attention can be attributed to its uprising prevalence in school settings, as a meta-analytic study estimated that 70–95% of students procrastinated problematically [2]. Previous studies have revealed that procrastinating behaviors are associated with various negative outcomes among students, including poor grades, loss of interest in the course, accumulated workloads that might subsequently predict emotional exhaustion, course withdrawal or even time wasting with self-handicapping behaviors [3]. From the perspective of psychological well-being, long-term procrastinating behaviors are associated with the presence of symptoms of stress, depression, and anxiety [4,5,6,7], feelings of shame and guilt [8,9,10] and negative mental health outcomes [11,12]. Also, college students’ procrastinating behaviors have been shown to predict their subsequent low levels of personal planning and life satisfaction in adulthood [13]. With these negative consequences of long-term procrastinating behaviors, psychologists have long attempted to provide possible explanations for procrastination.

In Asian societies that emphasize Confucianism (e.g., Hong Kong, China, and Korea), a high level of academic achievement is considered a prerequisite for future career success [14]. As a result, the education systems in these societies are highly competitive, and students are expected to outperform their peers in the college entrance exams to get admitted by prestigious universities [15,16]. Under these circumstances, academic comparisons are common [17]. A common form of comparison is termed “parents’ forced social comparison” (PFSC), which is characterized by parent-induced act of comparing their children’s academic performance to their superior aged-matched peers [15]. While PFSC may raise children’s academic striving behaviors (e.g., more hours spent on doing revisions), it can also be associated with students’ diverse aversive impacts [15,16]. For instance, according to the social rank theory, making social comparisons with other group members may lead to the propensity of perceiving themselves as inferior and agonizing their weaknesses [16,18]. Past studies have revealed that students who frequently experience or make social comparisons tend to develop a sense of inferiority, which in turn can lead to painful emotional experiences such as self-doubts and frustration, diminished subjective well-being, manifestation of signs of depression, harsh self-evaluations, and low self-esteem [19]. In this study, the authors examined how striving behavior derived from university students’ sense of inferiority may explain their procrastination.

### 1.1. Striving to Avoid Inferiority and Procrastination

Striving to avoid inferiority (SAI) refers to the tendency that people feel insecure and unsafe in their social relationships and therefore experience pressure to strive to avoid unwanted inferiority pertaining to rejection, criticism, being overlooked by others [20,21,22]. From a psychoanalytic perspective, the primary reason for developing a high degree of SAI is the failure to form a secure attachment, which makes people perceive their social places as insecure, unsafe, or easily lost, driving them to strive hard to be accepted and liked by other people [23]. Usually, competitive environments might enhance the competitive nature of people’s social relationships, and their focus of ranking, hierarchical and social mentality, which in turn intensify their social competitions and comparisons and raise their concerns about consequent rejection and inferiority [20,21,22]. As a result, their internal fear would be stimulated and they would strive to make a position in their social relationships to prevent being rejected, criticized, or overlooked by others and conceal their inferiority [20,21,22]. Given the similarity between SAI and perfectionistic concern, which is featured by an overly critical evaluation of oneself with reference to the discrepancy between one’s standard and actual performance and its association with difficulties in initiating an intended task (i.e., procrastination) [24], we thereby argued that SAI should be associated with procrastination. Also, since the underlying mechanism through which SAI influences procrastinating behaviors have not been well studied, the present study aimed at exploring and elucidating the mechanisms between these two constructs.

### 1.2. Procrastination as a Self-Regulatory Failure—Why Stress Matters?

Among the predictors of procrastination, self-control has attracted great attention from psychologists. Self-control refers to people’s ability to utilize their will-power and effortful cognitive process to moderate their behaviors [7]. In other words, self-control behavior is a controlled process which demands mental attention and cognitive resources and cannot occur in an automatic, effortless fashion [25]. Specifically, it is proposed that self-control involves setting goals, engaging in goal-directed behavior, monitoring task progress toward goals, and adjusting one’s behavior when sufficient progress towards the goals has not been made [26]. Behaviorally oriented psychologists have conceptualized procrastination as a main reason of failure to perform self-control [3,27,28,29]. Theoretically, according to the ego-depletion theory, human beings are thought to possess a limited capacity for self-control (i.e., limited self-control resource model) [30,31]. Specifically, performing any cognitively demanding and effortful task demands inhibition and attentional efforts, which directly drain their inner cognitive resources, as the individuals are required to override their general propensity to wander and avoid the stressful condition [32]. Laboratory experiments have supported this model, indicating that self-control consumes these cognitive resources [33]. Also, it has been argued that the need of self-monitoring would be stronger if the threat or stress level is persistent and uncontrollable [34].

Besides, peoples’ motivation to work or to procrastinate is largely dependent upon their emotional states. Yerkes–Dodson law proposed a curvilinear relationship between mental arousal and performance, that is, at some point of arousal (i.e., stress), performance would drop (rather than increase) as the arousal is increasing continually [35]. Regarded as one of the negative emotional experiences associated with biochemical, physiological, and behavioral changes, stress serves the purpose of dealing with stressful events [36]. A normal amount of stress level is beneficial and necessary for individuals’ motivation to perform tasks. Excessive amount of stress, however, can result in negative health outcomes which aversively influences the immune, cardiovascular, neuroendocrine, and central nervous systems [37,38,39]. Moreover, recent studies have found that exposure to psychosocial stress is associated with a range of impaired cognitive and affective functions including sustained attention, concentration, spatial, verbal working memory, and presence of depression and anxiety symptoms [40,41]. In the same vein of the ego-depletion theory, prolonged exposure to stress would also aversively impact individuals’ subsequent performance of self-regulatory behaviors, leading to the lack of self-control and disinhibition, which in turn lead to procrastination [30,31,42,43,44]. Therefore, it is believed that elevated stress level consumes inner attentional and cognitive resources and therefore attenuates individuals’ self-control, resulting in procrastination.

### 1.3. SAI and Procrastination, the Mediating Roles of Stress and Self-Control

A study has demonstrated that elevated degree of SAI exacerbates subjects’ vulnerability to stress [45]. This finding corroborates the social rank theory positing that the experience of feeling placed in unwanted inferior positions and being marginalized can increase negative affect and reduce positive affect [16,18,46]. SAI is associated with various negative outcomes, including daily stress, avoidant coping, low perceived social support, and negative affect [47]. According to Gilbert, when people fear the consequences of being inferior or subordinate to others, they would be driven to strive to prevent both self and others from evaluating the self as inferior [48]. Therefore, self-other relationships would be competitive rather than warm, caring, or cooperative [45]. Also, people with a high degree of SAI would concentrate on their social comparisons and shame sensitivities, and SAI is a source of elevated stress as individuals find it difficult to feel socially accepted and safe in their social relationships [21,49]. From the perspective of ego-depletion theory, exerting self-control behavior expends inner cognitive resources, which reduces the amount of resources available for subsequent self-control efforts [30,31]. Once people have to cope with their stress and the negative feelings, their inner cognitive resources will also be depleted, which impacts their subsequent performance of self-control behaviors [43,44,50]. The common impacts of self-control deficiency include irritation, regret, despair, self-blame, and more importantly, impaired academic and work progress [51]. Recently, it has been revealed that while the affective construct of depression and cognitive construct of perfectionism concern have moderate effects on procrastination among college students, those with difficulties in performing self-control also report high levels of maladaptive procrastinating behaviors, implying that the behavioral construct of self-control may be a strong indicator of subsequent procrastination [52,53]. In this vein, we proposed that stress and self-control might underpin the positive relationship between SAI and procrastination, and those with high SAI would be more likely to procrastinate more.

### 1.4. The Present Research

In summary, given the shared featured between SAI and perfectionistic concern, it was interesting to examine the relationship between university students’ SAI and procrastination, as well as the psychological mechanisms underlying these two constructs. Previous research has revealed that self-control is a strong predictor of procrastination [52]. Also, the essence of the ego-depletion theory is that both attempt at completing an effortful cognitive task and moderation of one’s emotion drain off the same pool of inner limited cognitive resources, and elevated level of stress can lead to lower successful self-regulatory behavior [54,55], it was therefore hypothesized that self-control would mediate the positive relationship between stress and procrastination (H1). Besides, given the corroborated positive relationship between SAI and elevated stress, it was also hypothesized that stress would mediate the negative relationship between SAI and self-control (H2). Finally, we hypothesized that stress and self-control would serially mediate the positive relationship between SAI and procrastination (H3). The present study intended to extend the existing knowledge by investigating the indirect processes underlying the relationship between SAI and procrastination.

## 2. Materials and Methods

### 2.1. Research Design and Participants

The study used a cross-sectional survey design. Participants were 154 Hong Kong undergraduate students from Hong Kong Shue Yan University (HKSYU; *n* = 96), Hong Kong University of Science and Technology (HKUST; *n* = 18), City University of Hong Kong (CityU; *n* = 24), and Hong Kong Baptist University (HKBU; *n* = 16). There were 42 first-year students, 47 second-year students, 39 third-year students, 26 fourth-year students. Undergraduate students were recruited via convenience sampling from June to August in 2016 (i.e., the summer academic semester of 2015–2016). Prior to providing consent to the study, all participants were informed of the nature of the research and the principles of confidentiality. The ages of the participants ranged from 16 to 45, with a mean age of 21.9 (*SD* = 3.37). Among the respondents, 66 were male and 88 were female. Further participants’ demographic information is shown in Table 1.

### 2.2. Measures

#### 2.2.1. General Procrastination Scale (GPS)

The GPS was used to measure participants’ severity of procrastination [56]. The scale contains 20 items rated on a 5-point Likert scale (1 = false for me, 5 = true for me) (e.g., I often find myself performing tasks that I had intended to do days before), including 10 reverse-scored items. Higher scores indicate higher levels of procrastination propensity. The internal consistency reliability Cronbach’s alpha for the 20 items was 0.84 in the present study.

#### 2.2.2. The Stress Subscale of the Depression Anxiety Stress Scales (DASS-21)

The DASS-21 is the shortened version of the DASS-42 [57]. This scale includes 21 items with three subscales assessing the levels of depression, anxiety and stress. The scale uses a 4-point Likert scale for participants to rate how much each statement applied to them over the past week (0 = did not apply to me at all, 3 = applied to me very much, or most of the time). In this study, only the stress subscale was used. A sample item of this subscale is, “I found it hard to wind down.” Higher scores indicate higher levels of stress over the past week. The stress subscale had good reliability, with an internal consistency reliability Cronbach’s alpha of 0.81 in the present study.

#### 2.2.3. Striving to Avoid Inferiority Scale (SAIS)

The SAIS is a 31-item scale of striving to avoid inferiority tendencies [46]. The scale consists of two subscales, including insecure striving and secure non-striving. Only the subscale of insecure striving was used in this study. A sample item of this subscale is, “If I don’t strive to achieve, I’ll be seen as inferior to other people.” Participants were asked to rate statements describing how they thought and felt about their needs to strive and compete in life. Higher scores indicate higher levels SAI. Each item is answered using a 5-point Likert scale (0 = never, 4 = always). The SAIS subscales had good reliability, with a Cronbach’s alpha of 0.91 for insecure striving in the present study.

#### 2.2.4. Short Self-Regulation Questionnaire (SSRQ)

This 31-item instrument measures generalized participants’ ability to control their behavior to achieve desired future outcomes [58]. Each item is answered using a 5-point Likert scale (1 = strongly disagree; 5 = strongly agree) (e.g., “I set goals for myself and keep track of my progress”). Higher scores indicate higher levels of self-control tendencies. The scale had a good reliability with Cronbach’s alpha of 0.82 in the present study.

### 2.3. Statistical Analyses

**Figure 1:**
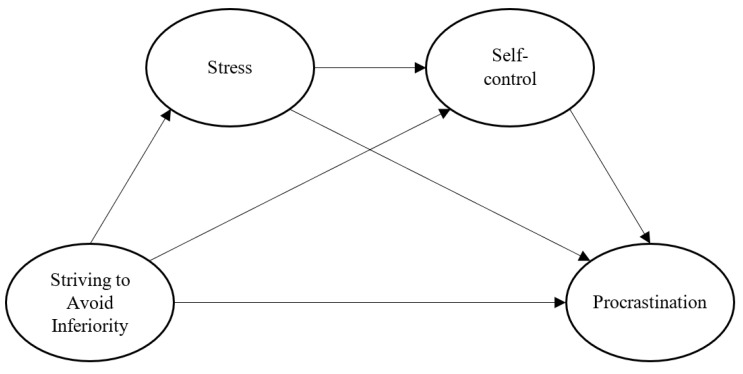
The hypothesized serial mediation model.

Structural equation modeling was conducted using the AMOS program to test the hypothesized relationships as shown in Figure 1. The significance of the indirect effect of stress on procrastination mediated by self-control, the indirect effects of SAI on self-control mediated by stress, and the indirect effect of SAI on procrastination mediated by stress and then self-control, were tested using the Sobel test and the bootstrapping technique.

## 3. Results

### 3.1. Descriptive Statistics and Correlational Analyses

The means, standard deviations and the correlations among SAI, stress, self-control, and procrastination were shown in the observed correlation matrix in Table 2. Correlational analyses revealed that SAI was positively related to stress, *r*(152) = 0.40, *p* < 0.001, stress was negatively related to self-control, *r*(152) = −0.32, *p* < 0.001, and self-control was negatively related to procrastination, *r*(152) = −0.53, *p* < 0.001. However, SAI was not significant associated with procrastination, *r*(152) = 0.04, *p* > 0.05, implying that no direct pathway predicting procrastination from the SAI tendency was observed.

### 3.2. Structural Equation Modeling

Structural equation modeling with maximum likelihood was conducted to analyze the hypothesized serial mediation model shown in Figure 1. Because of the small sample size relative to the number of items, the item parceling method was employed [59,60]. Three item parcels were formed for each scale. A satisfactory model fit was indicated by a root mean square error of approximation (RMSEA) < 0.06, a standardized root mean square residual (SRMR) < 0.08, a comparative fit index (CFI) > 0.95, and a Tucker-Lewis index (TLI) > 0.95 [61].

The hypothesized serial mediation model shown in Figure 1 was tested. The model achieved a satisfactory fit, χ^2^(48, *N* = 154) = 68.44, *p* = 0.028, RMSEA = 0.053, 90% CI [0.018, 0.080], SRMR = 0.049, CFI = 0.980, TLI = 0.973. The model was modified by removing three non-significant direct effects, including the direct effects from SAI and stress on procrastination, and the direct effect from SAI on self-control. The modified model showed an adequate fit, χ^2^(51, *N* = 154) = 75.33, *p* = 0.015, RMSEA = 0.056, 90% CI [0.025, 0.081], SRMR = 0.064, CFI = 0.977, TLI = 0.970. A chi-square difference test revealed that the deletion of the three direct effects did not result in a significant deterioration of the model fit, Δ χ^2^(3, *N* = 154) = 6.89, *p* = 0.076. Hence, the more parsimonious modified model was retained as the final model.

**Figure 2:**
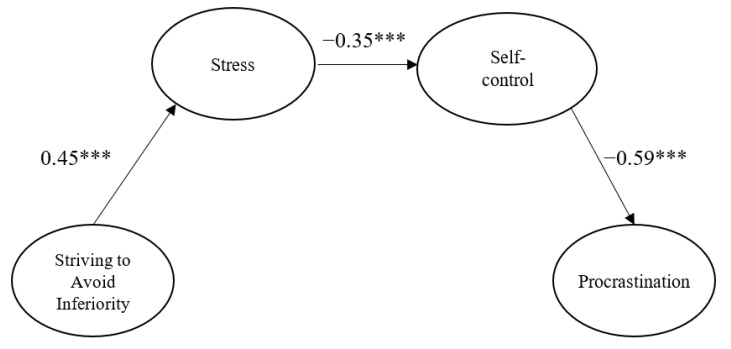
Standardized coefficients for the final model. Measurement errors and parceled indicators are omitted for clarity. *** *p* < 0.001.

The final model is illustrated in Figure 2. Standardized factor loadings of the parcels were high, ranging from 0.76 to 0.91. Procrastination was negatively predicted by self-control (β = −0.59, *p* < 0.001). Self-control was negatively predicted by stress (β = −0.35, *p* < 0.001). Stress was positively predicted by SAI (β = 0.45, *p* < 0.001).

The significance of the indirect effects was tested using the Sobel test and the bootstrapping technique. Biased corrected 95% confidence intervals (BC 95% CIs) were obtained based on 5000 bootstrap resamples. The indirect effect of SAI on self-control via stress was significant, (β = −0.16, *z* = −3.00, *p* = 0.003, BC 95% CI [−0.27, −0.08]). Moreover, the indirect effect of stress on procrastination via self-control was significant, (β = 0.21, *z* = 3.19, *p* = 0.001, BC 95% CI [0.10, 0.34]). Finally, the three-path indirect effect of SAI on procrastination via stress and self-control was significant (β = 0.10, *z* = 2.71, *p* = 0.007, BC 95% CI [0.05, 0.17]). Overall, these results were supportive of H1, H2 and H3 (see Figure 2).

## 4. Discussion

First, the present study supported the hypothesis (H1) that higher stress would positively predict procrastination mediated by self-control. This was in line with the previous studies noting that procrastination is regarded as a self-control failure [30,31]. According to the ego-depletion theory, both performing an effortful cognitive task and controlling one’s emotions may drain off the same pool of inner limited cognitive resources, and a high level of stress can predict poor subsequent self-regulatory behavior [54,55], and those who undesirably self-control themselves would tend to choose “myopic”, short-term benefits over long-term gains, and procrastinating behavior is a typical behavioral outcome of such propensity [27,28,52,62].

Besides, the present findings supported the hypothesis (H2) that stress would mediate the negative relationship between SAI and self-control. Individuals with elevated tendency of SAI would suffer from higher vulnerability to aversive affective states, including higher stress level [45]. Also, a meta-analysis on ego-depletion theory provided robust evidence that after a person has made one attempt at performing self-control, later attempts at self-control would be less likely to succeed, which in turn would undermine one’s ability to perform self-control behavior [33]. Researchers later have extended this theory and argued that when people experience elevated stress, they would need to regulate their mood or emotional states accompanied by stress to restore equilibrium for performing subsequent self-regulatory, controlled behavior [54,55]. Mood restoration requires the suppression of negative mood, and inhibition and self-control are therefore prerequisites [30,31]. This was consistent with the notion that individuals with such striving tendency are more likely to be trapped by prolonged exposure to an elevated level of stress, which would consume the inner cognitive resources available for maintaining subsequent self-regulatory behaviors, resulting in a decline in self-control [44,54,55,63,64].

More importantly, results of SEM in the present study supported the hypothesis (H3) that stress and self-control would serially mediate the effect of SAI on procrastination. The results indicated that SAI would indirectly lead to procrastination, through the underlying mechanisms of stress and self-control serially. The phenomenon of SAI is alike to the performance-avoidance goal orientation, which is defined as the tendency to strive to avoid poorer performance relative to others [65,66]. Previous research has found that performance-avoidance goal orientation is positively associated with procrastinating behavior in a way that individuals carry out maladaptive self-regulatory processes rooted in concerns about failure and inadequacy throughout the task processes, which might paradoxically increase their perceived task aversiveness [67].

The present study provided further evidence on the effect of striving on procrastination by demarcating SAI from “secure non-striving” (i.e., those do not intentionally strive for the fear of ostracism and rejection). Traditionally and intuitively, it was commonly believed that individuals who are motivated to strive for success were less prone to procrastination, given their concerns about achievements [46,68,69]. According to Gilbert and coworkers, SAI tendency can be considered as a defensive coping strategy (strategy that aims at preventing aversive consequence), which drives individuals to comply with rules and demands in absolute manners formulated by their disciplinary parents when they were young and had no right and power to bargain with them (e.g., being forced to accomplish all the assignments before watching television) [46]. Therefore, it is inferred that individuals under these circumstances would probably have better self-control and be less inclined to procrastinate because of their well-developed personality characteristics of conscientiousness, which are manifested by setting and keeping long-range goals, taking seriously obligations to self and others, and perfectionism through adhering rigidly to unrealistically high expectations and standards [68,69]. However, contrary to Gilbert and colleagues’ arguments [46], findings in the present study revealed that SAI would indirectly influence procrastination through stress and self-control. In short, the present study emphasized the aversive impacts of striving tendency originated from the fear of ostracism and social rejection.

### 4.1. Research Implications

The originality of the present study lied in the fact that SAI would indirectly predict procrastination among university students through the serial underlying mechanisms of stress and self-control, implying that high SAI tendency would result in elevated stress level, leading to high chance of developing problematic self-control and dilatory behaviors. While addressing university students’ academic motivational issues (including procrastination), teachers, tutors and other mental health practitioners should note this crucial but largely overlooked psychological dimension, for its potential associations with elevated stress level, self-control difficulties and procrastinating behaviors. One of the plausible intervention approaches is to promote students’ self-forgiveness, which not only alleviates negative affects but also makes students more motivated to accept their responsibility, thereby reducing their procrastinating behaviors [10].

### 4.2. Limitations and Further Research Directions

Despite the study’s important implications, the present study had its methodological limitations. Firstly, as this study used a cross-sectional design, cautions had to be made when drawing causal relationships between the psychological constructs stated above. Secondly, this study did not investigate the university students’ inner dynamic, ever-changing thoughts (psychological or cognitive processes) and feelings (emotional reactions) when they procrastinated some significant tasks, not least the pathways of trapping into procrastination and how they managed to get rid of procrastination when the assignment/exam deadlines were approaching. Hence, further studies on this topic can adopt qualitative research strategies so that researchers and practitioners may have better picture of the cycle of procrastination, which help them conduct early screening and identification of those with potential propensity of falling into the plight of procrastination for offering them better interventions.

## 5. Conclusions

In a nutshell, the present study conducted among Hong Kong university students supported the hypotheses that self-control mediates the relationship between stress and procrastination (H1), stress mediates the relationship between SAI and self-control (H2), and the relationship between SAI and procrastination is serially mediated by stress and self-control (H3). Further research may explore alternative models explaining the relationships between procrastination and attitudes relevant to striving, including but not limited to perfectionistic concerns, performance-oriented goal orientation, and conduct qualitative research to investigate the pathways or cycles of procrastination among university students. The findings in this study could expand our existing knowledge of psychological variables that would potentially lead to dilatory behaviors. Specifically, these findings revealed that striving behavior out of the fear of social rejection or sense of inferiority could be an early indicator of subsequent problems of self-control failure and procrastination.

## Figures and Tables

**Table 1:** Demographic characteristics of the student participants (N = 154).

Categorical Variables	n	%
Gender		
Male	66	42.9
Female	88	57.1
Mode of Study		
Full-time	143	92.9
Part-time	11	7.1
Year of Study		
1	42	27.3
2	47	30.5
3	39	25.3
4	26	16.9
Monthly Household Income		
<10,000	13	8.5
10,000–14,999	21	13.7
15,000–19,999	15	9.7
20,000–29,999	24	15.6
30,000–39,999	15	9.7
40,000–49,000	15	9.7
50,000–69,999	9	5.8
>70,000	6	3.9
No information	36	23.4
Marital Status		
Single	147	95.6
Married	5	3.2
Cohabitated	1	0.6
Others	1	0.6
University Attended		
HKSYU	96	62.3
HKUST	18	11.7
CityU	24	15.6
HKBU	16	10.4
Programme of Study		
Science	10	6.5
Engineering	15	9.7
Business/Economics/Finance	47	30.5
Social Science	82	53.3
**Continuous Variable**	**M**	**SD**
Age	21.9	3.37

**Table 2:** Means, Standard Deviations and the Intercorrelations among Variables of the Study.

Measure	Mean	SD	1	2	3
1. Stress	1.08	0.59	–		
2. Procrastination	3.01	0.58	0.06	–	
3. Insecure striving	2.27	0.69	0.40 ***	0.04	–
4. Self-control	3.20	0.39	−0.32 ***	−0.53 ***	−0.05

Note. *** *p* < 0.001.

## Data Availability

The data presented in this study are available on request from the corresponding author. The data are not publicly available due to privacy considerations.
